# Supplementary material for: CagA toxin and risk of Helicobacter pylori-infected gastric phenotype: A meta-analysis of observational studies
Source: PLoS One. 2024 Aug 22;19(8):e0307172. doi: 10.1371/journal.pone.0307172 (PMC11341061; doi:10.1371/journal.pone.0307172)
Supplement: S1 Table — (DOC) [file pone.0307172.s002.doc]

**S1 Table. Search strategy in PubMed**

(((("cagA"[All Fields] AND ("helicobacter pylori"[MeSH Terms] OR ("helicobacter"[All Fields] AND "pylori"[All Fields]) OR "helicobacter pylori"[All Fields])) OR ("helicobacter pylori"[MeSH Terms] OR ("helicobacter"[All Fields] AND "pylori"[All Fields]) OR "helicobacter pylori"[All Fields] OR "h pylori"[All Fields])) AND ("gastritis"[MeSH Terms] OR "gastritis"[All Fields] OR "gastritides"[All Fields])) OR ("peptic ulcer"[MeSH Terms] OR ("peptic"[All Fields] AND "ulcer"[All Fields]) OR "peptic ulcer"[All Fields])) AND ("stomach neoplasms"[MeSH Terms] OR ("stomach"[All Fields] AND "neoplasms"[All Fields]) OR "stomach neoplasms"[All Fields] OR ("gastric"[All Fields] AND "cancer"[All Fields]) OR "gastric cancer"[All Fields]) AND ("asia"[MeSH Terms] OR "asia"[All Fields])
