# Supplementary material for: CagA toxin and risk of Helicobacter pylori-infected gastric phenotype: A meta-analysis of observational studies
Source: PLoS One. 2024 Aug 22;19(8):e0307172. doi: 10.1371/journal.pone.0307172 (PMC11341061; doi:10.1371/journal.pone.0307172)
Supplement: S2 Table — (DOC) [file pone.0307172.s003.doc]

**S2 Table. Excluded studies and the reasons for exclusion**

| Study, yr | Justification | Citation |
| --- | --- | --- |
| Miftahussurur 2021 | Not primary data | Miftahussurur M, Waskito LA, Fauzia KA, et al. Overview of *Helicobacter pylori* infection in Indonesia: what distinguishes it from countries with high gastric cancer incidence? Gut Liver. 2021;15(5):653-665. |
| Miftahussurur 2015 | Small samples (less than 10) | Miftahussurur M, Shiota S, Suzuki R, et al. Identification of *Helicobacter pylori* infection in symptomatic patients in Surabaya, Indonesia, using five diagnostic tests. Epidemiol Infect 2015; 143:986-996 |
| Syam, 2015 | No data for *Cag*A | Syam AF, Miftahussurur M, Makmun D, et al. Risk Factors and Prevalence of *Helicobacter pylori* in Five Largest Islands of Indonesia: A Preliminary Study. PLoS One. 2015;10(11):e0140186. |
| Myint 2015 | No data for *Cag*A | Myint T, Shiota S, Vilaichone RK, et al. Prevalence of *Helicobacter pylori* infection and atrophic gastritis in patients with dyspeptic symptoms in Myanmar. World J Gastroenterol. 2015;21(2):629-36 |
| Nguyen, 2006 | Not assess *Cag*A | Nguyen BV, Nguyen KG, Phung CD, et al. Prevalence of and factors associated with *Helicobacter pylori* infection in children in the north of Vietnam. Am J Trop Med Hyg. 2006 ;74(4):536-9. . |
| Nguyen, 2021 | No data for gastric disorder | Nguyen TH, Ho TTM, Nguyen-Hoang TP, et al. The endemic *Helicobacter pylori* population in Southern Vietnam has both South East Asian and European origins. Gut Pathog. 2021 Sep 30;13(1):57. |
| Subsomwong, 2017 | No separate data for HP positive gastric disorders. | Subsomwong P, Miftahussurur M, Uchida T, et al. Prevalence, risk factors, and virulence genes of *Helicobacter pylori* among dyspeptic patients in two different gastric cancer risk regions of Thailand. PLoS One, 2017. 12(10): e0187113. |
| Sheikh, 2018 | Not from the targeted region | Sheikh AF, Yadyad MJ, Goodarzi H, et al. CagA and vacA allelic combination of *Helicobacter pylori* in gastroduodenal disorders. Microb Pathog. 2018 ;122:144-150 |
