# Supplementary material for: CagA toxin and risk of Helicobacter pylori-infected gastric phenotype: A meta-analysis of observational studies
Source: PLoS One. 2024 Aug 22;19(8):e0307172. doi: 10.1371/journal.pone.0307172 (PMC11341061; doi:10.1371/journal.pone.0307172)
Supplement: S3 Table — (DOC) [file pone.0307172.s004.doc]

**S3 Table. Quality assessment with the NOS criteria**

| Study | Year | Country | Selection | Comparability | Exposure/outcome | Total rating star |
| --- | --- | --- | --- | --- | --- | --- |
| Myint | 2018 | Myanmar | 3 | 2 | 3 | 8 |
| Ansari | 2017 | Myanmar | 3 | 2 | 1 | 6 |
| Jeyamani | 2018 | India | 3 | 2 | 2 | 7 |
| Linpisarn | 2007 | Thailand | 3 | 1 | 2 | 6 |
| Boonyanugomol | 2020 | Thailand | 3 | 1 | 2 | 6 |
| Nguyen | 2023 | Vietnam | 3 | 2 | 2 | 7 |
| Nguyen | 2010 | Vietnam | 3 | 2 | 2 | 7 |
| Pandya | 2017 | India | 3 | 1 | 1 | 5 |
| Chomvarin | 2008 | Thailand | 3 | 2 | 2 | 7 |
| Chomvarin | 2012 | Thailand | 3 | 1 | 2 | 6 |
| Yamada | 2013 | Thailand | 3 | 2 | 2 | 7 |
| Zheng | 2000 | Singapore | 3 | 1 | 2 | 6 |
| Trang | 2015 | Vietnam | 3 | 2 | 3 | 8 |
|  | 2015 | Myanmar | 3 | 2 | 3 | 8 |
| Uchida | 2009 | Thailand | 3 | 2 | 2 | 7 |
| Alfizah | 2012 | Malaysia | 3 | 2 | 3 | 8 |
| Mohamed | 2009 | Malaysia | 3 | 1 | 2 | 6 |
| Miftahussurur | 2015 | Indonesia | 3 | 1 | 1 | 5 |
| Tiwari | 2011 | India | 3 | 1 | 2 | 6 |
| Ali | 2005 | India | 3 | 1 | 2 | 6 |
| Tan | 2005 | Malaysia | 3 | 1 | 2 | 6 |
| Tan | 2006 | Malaysia | 3 | 2 | 2 | 7 |
| Truong | 2009 | Vietnam | 3 | 1 | 2 | 6 |
| Schmidt | 2009 | Malaysia | 3 | 1 | 2 | 5 |
| Schmidt | 2010 | Malaysia | 3 | 2 | 2 | 7 |
