# Supplementary material for: CagA toxin and risk of Helicobacter pylori-infected gastric phenotype: A meta-analysis of observational studies
Source: PLoS One. 2024 Aug 22;19(8):e0307172. doi: 10.1371/journal.pone.0307172 (PMC11341061; doi:10.1371/journal.pone.0307172)
Supplement: S2 Fig — (DOC) [file pone.0307172.s006.doc]

**S2 Fig. Pooled prevalence of CagA in *H. pylori-* infected peptic ulcer disease**

Note: A horizontal line represents individual study. A point estimate (prevalence) of individual study is represented by the square in the line. The 95% interval is indicated by the line's width. A pooled prevalence is represented by the diamond. Proportion is the estimated percentage of prevalence. The *I*^2^ value in percentage denotes the degree of heterogeneity between studies. Ali (2005), for instance, reported a prevalence of 51% (95%CI: 41-61%).
