# Supplementary material for: CagA toxin and risk of Helicobacter pylori-infected gastric phenotype: A meta-analysis of observational studies
Source: PLoS One. 2024 Aug 22;19(8):e0307172. doi: 10.1371/journal.pone.0307172 (PMC11341061; doi:10.1371/journal.pone.0307172)
Supplement: S3 Fig — (DOC) [file pone.0307172.s007.doc]

**S3 Fig. Pooled prevalence of CagA in *H. pylori-* infected gastric cancer**
