# Supplementary material for: CagA toxin and risk of Helicobacter pylori-infected gastric phenotype: A meta-analysis of observational studies
Source: PLoS One. 2024 Aug 22;19(8):e0307172. doi: 10.1371/journal.pone.0307172 (PMC11341061; doi:10.1371/journal.pone.0307172)
Supplement: Attachment — Submitted filename: PONE-D-24-13758 (1) MANUSCRIPT WITH REVIEW COMMENTS.docx [file pone.0307172.s008.docx]

**PLOS ONE**

### CagA toxin and risk of Helicobacter pylori-infected gastric phenotype: A meta-analysis of observational studies

--Manuscript Draft--

| **Manuscript Number:** | PONE-D-24-13758 |
| --- | --- |
| **Article Type:** | Research Article |
| **Full Title:** | CagA toxin and risk of Helicobacter pylori-infected gastric phenotype: A meta-analysis of observational studies |
| **Short Title:** | CagA toxin and risk of Helicobacter pylori-infected gastric phenotype |
| **Corresponding Author:** | Cho Naing, MBBS, PhD, FRCP James Cook University  Towns Valley, AUSTRALIA |
| **Keywords:** | Helicobacter pylori, gastric disorders, CagA, prevalence, risk factor, meta-analysis |
| **Abstract:** | Background  Helicobacter Pylori is frequently associated with non-cardia type gastric cancer, and it is designated as a group I carcinogen. This study aimed to determine the summary prevalence of cytotoxin-associated gene A (CagA) in people with Helicobacter pylori- infected gastric disorders and to investigate the relationship between CagA positivity and the risk of gastric disorders. This study focused on people in the Indo-Pacific countries.  Methods  Relevant studies were identified in the health-related electronic databases. For summary prevalence, meta-analysis of proportional studies was done, after Freeman- Tukey double arcsine transformation of data. A random-effect model was used to compute the summary odds ratio (OR) and 95% Confidence Interval (CI) to investigate the relationship between CagA and gastric disorder.  Results  Twenty-four studies from eight Indo-Pacific countries (Bhutan, India, Indonesia, Malaysia, Myanmar, Singapore, Thailand, Vietnam) were included. Overall, CagA positives in H. pylori-infected gastric disorders was 83% (73-91%,24 studies).  Following stratification, the pooled prevalence of CagA positives was 78% (95%CI:67- 90%,23 studies) in H. pylori-infected gastritis, 86% (95%CI:73-96%,17 studies) in peptic ulcer disease, and 83% (95%CI: 51-100%, 11 studies) in gastric cancer.  Geographic locations encountered variations in CagA prevalence. Compared with gastritis, there was a greater risk of developing gastric cancer in those with CagA positivity (OR:2.53,95%CI:1.15-5.55,7 studies).  Conclusion  Findings suggest that the distribution of CagA in H. pylori-infected gastric disorders varies throughout the eight Indo-Pacific countries, and CagA has a role in the development of gastric cancer. To confirm these findings, well-designed studies with sufficient samples are recommended. |
| **Order of Authors:** | Cho Naing, MBBS, PhD, FRCP |
|  | Htar Htar Aung |
|  | Saint Nway Aye |
|  | Yong Poovorawan |
|  | Maxine A Whittaker |
| **Additional Information:** |  |
| **Question** | **Response** |
| **Financial Disclosure** | The author(s) received no specific funding for this work. |

*Powered by Editorial Manager® and ProduXion Manager® from Aries Systems Corporation*

| Enter a financial disclosure statement that describes the sources of funding for the work included in this submission. Review the [submission guidelines](http://journals.plos.org/plosone/s/submission-guidelines#loc-financial-disclosure-statement) for detailed requirements. View published research articles from [*PLOS ONE*](https://journals.plos.org/plosone/) for specific examples.  This statement is required for submission and **will appear in the published article** if the submission is accepted. Please make sure it is accurate. |  |
| --- | --- |
| **Funded studies**  Enter a statement with the following details:   - Initials of the authors who received each award - Grant numbers awarded to each author - The full name of each funder - URL of each funder website - Did the sponsors or funders play any role in the study design, data collection and analysis, decision to publish, or preparation of the manuscript? |  |
| Did you receive funding for this work? |  |
| **Competing Interests**  Use the instructions below to enter a competing interest statement for this submission. On behalf of all authors, disclose any [competing interests](http://journals.plos.org/plosone/s/competing-interests) that could be perceived to bias this work—acknowledging all financial support and any other relevant financial or non- financial competing interests.  This statement is required for submission and **will appear in the published article** if the submission is accepted. Please make sure it is accurate and that any funding sources listed in your Funding Information later in the submission form are also declared in your Financial Disclosure statement.  View published research articles from | The authors have declared that no competing interests exist. |

*Powered by Editorial Manager® and ProduXion Manager® from Aries Systems Corporation*

| [*PLOS ONE*](http://journals.plos.org/plosone/) for specific examples. |  |
| --- | --- |
| **NO authors have competing interests**  Enter: *The authors have declared that no competing interests exist.*  **Authors with competing interests**  Enter competing interest details beginning with this statement:  *I have read the journal's policy and the authors of this manuscript have the following competing interests: [insert competing interests here]* |  |
| * typeset |  |
| **Ethics Statement**  Enter an ethics statement for this submission. This statement is required if the study involved:   - Human participants - Human specimens or tissue - Vertebrate animals or cephalopods - Vertebrate embryos or tissues - Field research   Write "N/A" if the submission does not require an ethics statement.  General guidance is provided below. Consult the [submission guidelines](http://journals.plos.org/plosone/s/submission-guidelines#loc-guidelines-for-specific-study-types) for detailed instructions. **Make sure that all information entered here is included in the Methods section of the manuscript.** | N/A |

*Powered by Editorial Manager® and ProduXion Manager® from Aries Systems Corporation*

| **Format for specific study types**  **Human Subject Research (involving human participants and/or tissue)**   - Give the name of the institutional review board or ethics committee that approved the study - Include the approval number and/or a statement indicating approval of this research - Indicate the form of consent obtained (written/oral) or the reason that consent was not obtained (e.g. the data were analyzed anonymously)   **Animal Research (involving vertebrate animals, embryos or tissues)**   - Provide the name of the Institutional Animal Care and Use Committee (IACUC) or other relevant ethics board that reviewed the study protocol, and indicate whether they approved this research or granted a formal waiver of ethical approval - Include an approval number if one was obtained - If the study involved *non-human primates*, add *additional details* about animal welfare and steps taken to ameliorate suffering - If anesthesia, euthanasia, or any kind of animal sacrifice is part of the study, include briefly which substances and/or methods were applied   **Field Research**  Include the following details if this study involves the collection of plant, animal, or other materials from a natural setting:   - Field permit number - Name of the institution or relevant body that granted permission |  |
| --- | --- |
|  |  |
| **Data Availability**  Authors are required to make all data underlying the findings described fully available, without restriction, and from the time of publication. PLOS allows rare exceptions to address legal and ethical concerns. See the [PLOS Data Policy](http://journals.plos.org/plosone/s/data-availability) and [FAQ](http://journals.plos.org/plosone/s/data-availability#loc-faqs-for-data-policy) for detailed information. | Yes - all data are fully available without restriction |

*Powered by Editorial Manager® and ProduXion Manager® from Aries Systems Corporation*

| A Data Availability Statement describing where the data can be found is required at submission. Your answers to this question constitute the Data Availability Statement and **will be published in the article**, if accepted. |  |
| --- | --- |
| **Important:** Stating ‘data available on request from the author’ is not sufficient. If your data are only available upon request, select ‘No’ for the first question and explain your exceptional situation in the text box. |  |
| Do the authors confirm that all data underlying the findings described in their manuscript are fully available without restriction? |  |
| **Describe where the data may be found in full sentences. If you are copying our sample text, replace any instances of XXX with the appropriate details.**   - If the data are **held or will be held in a public repository**, include URLs, accession numbers or DOIs. If this information will only be available after acceptance, indicate this by ticking the box below. For example: *All XXX files are available from the XXX database (accession number(s) XXX, XXX.*). - If the data are all contained **within the manuscript and/or Supporting Information files**, enter the following: *All relevant data are within the manuscript and its Supporting Information files.* - If neither of these applies but you are able to provide **details of access elsewhere**, with or without limitations, please do so. For example:   *Data cannot be shared publicly because of [XXX]. Data are available from the XXX Institutional Data Access / Ethics Committee (contact via XXX) for researchers who meet the criteria for access to confidential data.*  *The data underlying the results presented in the study are available from (include the name of the third party* | All relevant data are within the manuscript and its Supporting Information files. |

*Powered by Editorial Manager® and ProduXion Manager® from Aries Systems Corporation*

| *and contact information or URL).*   - This text is appropriate if the data are owned by a third party and authors do not have permission to share the data.   * typeset |  |
| --- | --- |
| Additional data availability information: |  |

*Powered by Editorial Manager® and ProduXion Manager® from Aries Systems Corporation*

1

1 **CagA toxin and risk of *Helicobacter pylori-*infected gastric**

# 2 phenotype: A meta-analysis of observational studies

3

4	Cho Naing^1¶^, Htar Htar Aung^2¶^, Saint Nway Aye^2^, Yong Poovorawan^3^, Maxine A Whittaker^1^

5

6 ^1^ College of Public Health, Medical and Veterinary Sciences, James Cook
7 University, Queensland, Australia
8 ^2^ School of Medicine, IMU University, Kuala Lumpur, Malaysia
9 ^3^ Center of Excellence in Clinical Virology, Department of Pediatrics, Faculty of
10 Medicine, Chulalongkorn University, Bangkok, Thailand

11

12	**^¶^**HHA and CN: Equal contributors and joint corresponding author.

13

14 **Corresponding author:**
15 Email: [cho3699@gmail.com](mailto:cho3699@gmail.com) (CN)
16 [htar.ng@gmail.com](mailto:htar.ng@gmail.com) (HHA)

17

18

19

20

21

22

23

# 24 Abstract

## 25 Background

26 *Helicobacter Pylori* is frequently associated with non-cardia type gastric cancer, and it is
27 designated as a group I carcinogen. This study aimed to determine the summary prevalence
28 of cytotoxin-associated gene A (CagA) in people with *Helicobacter pylori-*infected *gastric*
29 disorders and to investigate the relationship between CagA positivity and the risk of gastric
30 disorders. This study focused on people in the Indo-Pacific countries.

31

## 32 Methods

33 Relevant studies were identified in the health-related electronic databases. For summary
34 prevalence, meta-analysis of proportional studies was done, after Freeman-Tukey double
35 arcsine transformation of data. A random-effect model was used to compute the summary
36 odds ratio (OR) and 95% Confidence Interval (CI) to investigate the relationship between
37 CagA and gastric disorder.

38

## 39 Results

40 Twenty-four studies from eight Indo-Pacific countries (Bhutan, India, Indonesia, Malaysia,
41 Myanmar, Singapore, Thailand, Vietnam) were included. Overall, CagA positiv~~es~~ity in *H.*
42 *pylori-*infected gastric disorders was 83% (73-91%,~~24 studies~~). Following stratification, the
43 pooled prevalence of CagA positives was 78% (95%CI:67-90%,~~23 studies~~) in *H. pylori*-
44 infected gastritis, 86% (95%CI:73-96%,~~17 studies)~~ in peptic ulcer disease, and 83% (95%CI:
45 51-100%, ~~11 studies~~) in gastric cancer. Geographic locations encountered variations in CagA
46 prevalence. Compared with gastritis, there was a greater risk of developing gastric cancer in
47 those with CagA positivity (OR:2.53,95%CI:1.15-5.55,~~7 studies~~).

## 48 Conclusion

49 Findings suggest that the distribution of CagA in *H. pylori-*infected gastric disorders varies
50 throughout the eight Indo-Pacific countries, and CagA has a role in the development of
51 gastric cancer. To confirm these findings, well-designed studies with sufficient samples are
52 recommended in future.

53

54 **Keywords:** *Helicobacter pylori*, gastric disorders, CagA, prevalence, risk factor, meta-
55 analysis

56

57

58

59

60

61

62

63

64

65

66

67

68

69

70

# 71 Introduction

72 *Helicobacter pylori* is a gram-negative bacterium that colonizes the stomach and is attributed
73 to causing infections in humans [1.2]. As *H. Pylori* is frequently associated with non-cardia
74 type of gastric cancer, and it is designated as a group I carcinogen [3]. Studies reported that only
75 20% of the colonizing bacteria at any particular time directly interact with the stomach
76 epithelial cells [4],^4^ which ~~is~~ are insufficient to cause gastric disorder [5]. This reflects that, in
77 addition to its urease activity, *H. pylori* possesses additional virulence factors that are
78 required to enable it to colonize and survive in the stomach's acid environment. The
79 underlying mechanism of infectious diseases is greatly impacted by strain variations, which
80 could account for the different outcomes of *H. pylori* infection. Studies have documented that
81 *H. pylori* bacterial strains exhibit significant genetic diversity. Such diversity could result in
82 an array of clinical outcomes, including gastritis, peptic ulcer disease, gastric adenocarcinoma
83 (gastric cancer), and lymphoma. These outcomes depend on a variety of factors, particularly
84 the virulence determinants expressed by distinct strains of *H.pylori* [6,7]. Studies have
85 reported that virulent determinants such as cytotoxin-associated gene A (CagA), lipopoly-
86 saccharide, vacuolating cytotoxin, peptidoglycan, and gamma-glutamyl transpeptidase are
87 associated with gastric disease [8]. Among these, CagA is expressed by the terminal *cag*A
88 gene of the type IV secretion system (T4SS), and discovered on the cag pathogenicity island
89 (cagPAI) [9]. T4SS specifically forms a needle-like pilus device that is used to inject
90 virulence factor such as the CagA effector protein into host target cells [10-13]. *H. pyloria*
91 strains with CagA-positive are more virulent than CagA-negative bacteria because they are
92 linked to severe gastric inflammation [14].
93 With regards to gastric phenotype, there is an enigma coined as the "Asian Enigma"
94 [15] or "Asian Paradoxical" [16] that affects many Asian countries. This is because *H. pylori*
95 prevalence is particularly high in Thailand, Vietnam, India, and Bangladesh, while gastric
96 cancer prevalence is relatively low in these countries [17-21]. It draws attention to the need to
97 study whether CagA prevalence is related to this paradoxical pattern. Some studies assessed
98 CagA frequency in gastric disorders, which showed different findings. Since *H. pylori-*
99 infected patients showed variations in the clinical manifestations such as gastritis, peptic
100 ulcers, and gastric cancer, reporting the CagA prevalence on a range of gastric disorders is of
101 immense value. Taken together, the objectives of this study were to determine the pooled
102 prevalence of CagA status in populations ~~in~~ of the Indo-Pacific region with gastric disorders and
103 to investigate the association of CagA positive in the risk of gastric disorders.
104 104

# 105 Materials and Methods

106 This study sticked to the PRISMA-2020 checklist [22] [S1 Checklist]. The protocol of this
107 study is available from the corresponding author upon reasonable request. The consent from
108 the participants was not necessary as the current study used solely published data. There was
109 no funding to this study.
110 110

## 111 Search strategy

112 Two investigators (CN, HHA) looked at the relevant studies in health-related electronic
113 databases including PubMed, Ovid, Medline, Ovid Embase, Index Medicus, and Google
114 Scholar. The search terms used were (*Helicobacter pylori*, *H.pylori*, virulence factor, CagA,
115 gastric cancer, gastritis, and peptic ulcer) with appropriate Boolean operators (AND/OR). The
116 search was confined to publications in English between January 1, 2000, and November 18,
117 2023. Furthermore, hand-searching took place following the same inclusion criteria set for
118 this study. Search Strategy in PubMed is provided in S1 Table.

## 119 Selection criteria

120 Studies were considered if they met the following criteria.
121 1. Human studies of observational design that assessed adults with *H. pylori*-infected gastric
122 disorder.
123 2. Conducted in low- and middle-income Indo-Pacific countries.
124 3. Assessed the CagA status.
125 4. Provide sufficient data to compute the prevalence of CagA, and/or association of CagA
126 with any or all three gastric disorders (i.e., gastritis, peptic ulcer disease, gastric cancer)
127 5. Examined the presence of genotypes with the use of PCR.
128 6. Included a minimum of ten samples.
129 In this study, the participants with duodenal and/or gastric ulcerations were
130 categorised as peptic ulcer disease for estimation. Endoscopic gastritis was regarded as an
131 equivalent to ‘non-ulcer disorder’ with neither a history of ulcer disease nor endoscopic
132 evidence of ulcer disease [23].
133 133

## 134 Exclusion criteria

135 Studies that did not satisfy the criteria for inclusion were not considered. ~~Thus,~~ In addition, reviews, letters, editorials, efficacy trials, and modelling studies were omitted.
136 137

## 137 Data collection

138 One investigator (CN) screened the title and abstracts, which were deemed eligible. Two
139 investigators (HHA, CN) independently selected studies from those saved from the databases.
140 Full-text studies that were considered appropriate were further reviewed. Any deviation
141 between the two investigators was settled by agreement. Using a self-created data extraction
142 sheet, one investigator (CN) gathered data from the eligible studies. Another investigator
143 (HHA) cross-checked this. Data collection included first author, year of publication, study
144 country, study design, prevalence (numerator and denominator), participant characteristics
145 (age, sex), types of gastric disorders (gastritis, peptic ulcer, gastric cancer), and methods of
146 DNA sequencing.

## 147 Assessment of the quality of studies

148 The methodological quality of the studies involved in this study was rated using the
149 Newcastle-Ottawa scale (NOS) checklist [24]. ^T^his tool is a star rating scheme for
150 observational studies, where nine stars are the maximum to obtain. The higher the star
151 obtained, the better quality of the study.

## 152 Data analysis

153 At the individual-study level, the prevalence (along with variance) of CagA in a certain
154 gastric phenotype was estimated by dividing the number of CagA-positive cases by the total
155 number of cases examined. For pooling of studies, a proportional meta-analysis was carried
156 out as described by Barker and associates [25], after variance normalization with the
157 Freeman-Tukey double arcsine transformation [26]. For the association of CagA and the
158 development of gastric disorder, a pooled odds ratio (OR) with 95% CI was estimated.
159 Concerning the variations among studies, the DerSimonian and Laird random effect
160 model was employed for computing summary estimates [27]. An *I*^2^ value of >75% denotes
161 substantial variation [28]. Publication bias was not assessed for proportional meta-analysis
162 owing to a methodological shortcoming in Egger's tests and funnel plots [25]. As there was a
163 sufficient number of studies, Egger’s test was used to gauge publication bias for the risk
164 comparisons between gastritis and peptic ulcers. A simple test of asymmetry of the funnel
165 plot (plot of effect estimates against sample size) was visualised [29].
166 Data analyses were done with the “*metaprop_one”* command for proportional
167 meta-analysis, “*metan”* command for binary associations, and “*meta”* for publication bias
168 command in STATA (version 15) (TXT, USA).
169 170

# 170 Results

171 Fig 1 illustrates the PRISMA flow diagram indicating the study selection process in the
172 present meta-analysis. A total of 24 studies (26 datasets) were identified across eight Indo-
173 Pacific countries such as Bhutan, India, Indonesia, Malaysia, Myanmar, Thailand, Singapore,
174 and Vietnam [30-53]. Publication years spanned from 2005 [46] to 2023 [42]. A list of
175 excluded studies along with their justifications is provided in S2 Table.
176 177

### 177 Fig 1. PRISMA flowchart indicating the study selection process.

178 179
179 Table 1 presents the main characteristics of the 24 studies included in the current
180 meta-analysis. Most of the studies were conducted in Thailand (6 studies) and Malaysia (6
181 studies). These individual studies examined on a variety of gastric disorders, the most
182 common being gastritis (23 studies), followed by peptic ulcer disease (17 studies), and gastric
183 cancer (11 studies). The Trang study reported data from three countries, Bhutan, Myanmar
184 and Vietnam [49]. Fifteen studies reported data on comparison of CagA positive and
185 negatives in gastric disorders [31, 33-37,41-46, 49, 52,53]. The methodological quality of the
186 studies identified was a median of 6 stars (range 5-8 stars), reflecting most of the studies were
187 of moderate quality (S3 Table).
188 189
189 190
190 191

### 191 Table 1. Characteristics of the studies identified (N = 24)

192 193

| N  o. | Study | Yr | R  ef # | Count ry | Desi gn | Mean age, yr | Mal e n(%  ) | To tal | CagA+ve/tested samples | | | Remark |
| --- | --- | --- | --- | --- | --- | --- | --- | --- | --- | --- | --- | --- |
|  |  |  |  |  |  |  |  |  | GT | PU  D | GC |  |
| 1 | Myint | 20  18 | 4  0 | Myan  mar | CS | 40.1±1  1. | 28  (57) | 69 | 61/6  9 | NA | NA | 3DU+1  GC |
| 2 | Ansari | 20  17 | 3  2 | Myan mar | CS | 40.7±  11.5 | 30  (24) | 72 | 67/7  2 | NA | NA | GT 89.3%;  a subset data |
| 3 | Jeyamani | 20  18 | 3  6 | India | CS | NA | NA | 61 | 16/3  5 | 12/1  4 | 0/0 |  |
| 4 | Linpisarn | 20  07 | 3  7 | Thaila  nd | CS | NA | NA | 13  5 | 48/5  8 | 68/7  3 | 3/4 |  |
| 5 | Boonyanu gomol | 20  20 | 3  3 | Thaila nd | CS | NA | NA | 80 | 38/5  0 | 17/2  0 | 8/1  0 |  |
| 6 | Nguyen | 20  23 | 4  2 | Vietn  am | CS | 9.1±  2.4 | NA | 26  9 | 0 | 185 | NA |  |
| 7 | Nguyen | 20  10 | 4  1 | Vietn  am | CS | NA | NA | 10  0 | 71 | 24 | NA |  |
| 8 | Pandya | 20  17 | 4  3 | India | CS | R:10-  90 | NA | 43 | 9/34 | 6/9 | NA |  |
| 9 | Chomvari n | 20  08 | 3  4 | Thaila nd | CS | 49.5 | 55  (49) | 11  2 | 60/6  2 | 34/3  4 | 16/  16 |  |
| 1  0 | Chomvari n | 20  12 | 3  5 | Thaila nd | CS | 50 (R:  15-81 | 77  (52) | 14  7 | 65/6  8 | 52/5  7 | 17/  18 | Mixed/ unclassi fied not include  d. |
| 1  1 | Yamada | 20  13 | 5  2 | Thaila nd | CS | 56 ±  11.3^1^ | 93(  42) | 22  0 | 34/1  34 | NA | 7/8  6 | Benign  =GT; 1= GC  group |
| 1  2 | Zheng | 20  00 | 5  3 | Singa  pore | CS | NA | NA | 10  8 | 37/4  1 | 58/6  7 | NA |  |
| 1  3 | Trang | 20  15 | 4  9 | Vietn  am | CS | 44.1 ±  12.7 | 47  (46) | 10  2 | 72/7  6 | 25/2  6 | NA |  |
|  |  |  |  | Myan  mar | CS | 40.1 ±  11.7 | 28  (41) | 66 | 59/6  6 | NA | NA |  |
|  |  |  |  | Bhuta  n | CS | 36.8 ±  13.4 | 94  (47) | 20  0 | 161/  161 | 38/3  8 | NA |  |
| 1 | Uchida | 20 | 5 | Thaila | CS | 45 | 47 | 10 | 98 | NA | NA | 25=PU |

| 4 |  | 09 | 1 | nd |  |  | (45) | 3 |  |  |  | D |
| --- | --- | --- | --- | --- | --- | --- | --- | --- | --- | --- | --- | --- |
| 1  5 | Alfizah | 20  12 | 3  0 | Malay sia | CS | 56.05  ± 12.95 | 58  (53) | 11  0 | 81/8  1 | 28/2  8 | NA |  |
| 1  6 | Mohamed | 20  09 | 3  9 | Malay sia | CS | NA | NA | 93 | 44/9  3 | 26/9  3 | NA | Based on the total  tested. |
| 1  7 | Miftahuss urur | 20  15 | 3  8 | Indon esia | CS | 47.8 ± 14.6 | NA | 44 | 43 | 0 | 0 | 8 Other types are include  d. |
| 1  8 | Tiwari | 20  11 | 4  8 | India | CS | NA | NA | 32 | 3 | 15 | 14 |  |
| 1  9 | Ali | 20  05 | 3  1 | India | CS | 48.4 | 100  (57) | 17  4 | 4/56 | 53/1  04 | 12/  14 |  |
| 2  0 | Tan | 20  05 | 4  6 | Malay sia | CS | 55.53  ± 12.52 | 72(  57) | 12  7 | 59/7  5 | 25/2  8 | 0/2 |  |
| 2  1 | Tan | 20  06 | 4  7 | Malay sia | CS | 53.38  ± 12.09 | 36  (49) | 73 | 58/7  3 | NA | NA |  |
| 2  2 | Truong | 20  09 | 5  0 | Vietn  am | CS | NA | NA | 22 | NA | 11/1  1 | 11/  11 |  |
| 2  3 | Schmidt | 20  09 | 4  4 | Malay  sia | CS | NA | NA | 12  7 | 105/  105 | NA | 21/  21 |  |
| 2  4 | Schmidt | 20  10 | 4  5 | Malay  sia | CS | NA | 87  (55) | 15  9 | 75/7  5 | 16/1  6 | 22/  22 |  |

193 DU: duodenal ulcer; GC: gastric cancer; GT: gastritis; PUD: peptic ulcer disease;
194 **Prevalence of CagA in gastric disorders**
195 The CagA prevalence in *H. pylori-*infected gastric disorders was assessed in 24
196 studies, irrespective of the specific type (Fig 2). The overall pooled prevalence of CagA in
197 *H.pylori*-infected gastric disorders was 83% (73-91%). This indicated that the great majority
198 of population in Indo-Pacific region who had *H. pylori-*infected gastric disorders were CagA
199 positive. On stratification, Vietnam (90%, 95%CI: 73-99%; 4 studies) had the highest pooled
200 prevalence of CagA, followed by Malaysia (81%, 95%CI: 68-91%; 6 studies), Thailand

202	(81%, 95%CI: 48-99%; 6 studies), Myanmar (90%, 95%CI: 85-93%; 3 studies), and India

203 (60%, 95%CI: 34-84%; 4 studies). Notably, all except the studies in Myanmar (*I*^2^ :0%).
204 showed substantial heterogeneity (*I*^2^ :> 93%). There was only a single study from Bhutan

205	(100%, 95%CI: 98-100%), Indonesia (98%, 95%CI: 88-100%) or Singapore (88%, 95%CI:

206	80-93%).

207

### 208 Fig 2. Pooled prevalence of CagA in *H. pylori-*infected gastric disorders

209

## 210 CagA prevalence in *H.pylori-*infected gastritis

211 On stratification by type of gastric disorders, CagA prevalence in *H.pylori-*infected
212 gastritis was reported in 23 studies. Overall, the summary prevalence of CagA in gastritis was
213 79% (95%CI: 67-90%). Vietnam (94%, 95%CI: 90-97%; 2 studies) had the highest pooled
214 prevalence, followed by Malaysia (88%, 95%CI: 69-99%; 6 studies), Thailand (82%, 95%CI:

215	56-98%; 6 studies), Myanmar (88%, 95%CI: 82-93%; 2 studies), and India (20%, 95%CI: 6-

216 40%; 4 studies). Heterogeneity was substantial among studies in Malaysia and Thailand (*I*^2^: >
217 96%), but it was absent among studies in India, Myanmar, and Vietnam. Only a single study
218 was available either from Bhutan (100%, 95%CI: 98-100%), Indonesia (98%, 95%CI: 88-

219	100%) or Singapore (90%,95%CI: 77-97%) (S1 Fig).

220

## 221 CagA prevalence in *H.pylori-*infected peptic ulcer disease

222 Seventeen studies reported data on CagA prevalence among participants with *H. pylori*-
223 infected peptic ulcer disease. Overall, the summary prevalence of CagA in participants with
224 *H.pylori-*infected peptic ulcer disease was 86% (95%CI: 73-96%). The highest summary
225 prevalence of CagA was reported from Thailand 94% (95%CI: 87-99%; 4 studies), followed

226	by Vietnam (91%, 95%CI:50-100%; 4 studies) and Malaysia (86%, 95%CI: 37-100%, 4

227 studies). India accounted for a prevalence of 59% (95%CI: 43-75%; 4 studies). Heterogeneity
228 was substantial among these studies in India, Malaysia, Thailand, and Vietnam (*I*^2^: >57%). A
229 single study either from Bhutan (100% (95%CI: 91-100%) or Singapore (87%, 95%CI: 76-
230 94%) reported a prevalence of CagA in participants with peptic ulcer disease (S2 Fig).
231 231

## 232 CagA prevalence in *H.pylori-*infected gastric cancer

233 A total of 11 studies reported data on CagA prevalence among participants with *H. pylori-*
234 infected gastric cancer. Overall, 83% (95%CI:51-100%, *I*^2^: 95%) of patients with gastric
235 cancer had CagA positives. On stratification by countries, Malaysia had the highest
236 prevalence of CagA (96%, 95%CI: 87-100%, *I*^2^: 0%; 2 studies), followed by India (90%,

237	95%CI: 75-99%; *I*^2^: 0%; 2 studies), and Thailand (71%, 95%CI: 16-100%%; *I*^2^: 96%; 5

238 studies). Notably, heterogeneity was substantial among studies in Thailand (*I*^2^ > 98%), while
239 studies in India and Malaysia had the absence of heterogeneity. A single study was available

240	from Vietnam (92%, 95%CI: 65-99%) (S3 Fig).

241

## 242 Associations between CagA toxin and the risk of gastric disorder

243 Table 2 provides data on the association studies. Fifteen studies were available for an
244 assessment of the relationship between CagA and gastric disorder [31,33-37,41-46,49,52,53].
245 Of these, 13, seven, and six studies compared the risk between peptic ulcer disorder and
246 gastritis, gastric cancer and gastritis, and peptic ulcer disorder and gastric cancer,
247 respectively.
248 248
249 249
250 250
251 251

### 252 Table 2. Distribution of CagA in gastric phenotype

253 253

| **Sr.**  **no.** | **Study** | **Year** | **Ref#** | **Country** | **GT+**  **(total)** | **PUD+(total)** | **GC+(total)** |
| --- | --- | --- | --- | --- | --- | --- | --- |
| 1 | Ali | 2005 | 31 | India | 4 (56) | 49 (104) | 12(14) |
| 2 | Boonyanugomol | 2020 | 33 | Thailand | 38 (50) | 17 (20) | 8 (18) |
| 3 | Chomvarin | 2008 | 34 | Thailand | 60 (62) | 34 (68) | 16 (32) |
| 4 | Chomvarin | 2012 | 35 | Thailand | 65 (68) | 52 (109) | 17 (35) |
| 5 | Jeyamani | 2018 | 36 | India | 21 (42) | 12 (14) | NA |
| 6 | Linpisarn | 2007 | 37 | Thailand | 48 (58) | 68 (73) | 3 (7) |
| 7 | Nguyen | 2010 | 41 | Vietnam | 71 (76) | 24 (24) | NA |
| 8 | Nguyen | 2023 | 42 | Vietnam | NA | 185 (268) | NA |
| 9 | Pandya | 2017 | 43 | India | 9 (34) | 3 (12) | NA |
| 10 | Schmidt | 2009 | 44 | Malaysia | 103  (105) | NA | 21 (42) |
| 11 | Schmidt | 2010 | 45 | Malaysia | 121  (121) | 16 (48) | 22 (42) |
| 12 | Tan | 2005 | 46 | Malaysia | 59 (75) | 25 (53) | NA |
| 13 | Trang | 2015 | 49 | Vietnam | 72 (76) | 25 (51) | NA |
|  |  |  |  | Bhutan | 161(161) | 38 (38) | NA |
| 14 | Yamada | 2013 | 52 | Thailand | 71 (76) | 24 (48) | NA |
| 15 | Zheng | 2000 | 53 | Singapore | 58 (77) | 37 (78) | NA |

254 GC: gastric cancer; GT: gastritis; NA: not available/ not reported; PUD: peptic ulcer disease.
255 Thirteen studies were available for a comparison of the CagA positive between peptic
256 ulcer disease and gastritis risks [31-37, 41, 43-46, 49, 52, 53]. Only six studies were available
257 for a comparison of the CagA positive between gastric cancer and gastritis [33-35, 37, 44,
258 45]. Five studies reported data for all three types of gastric disorder [33-35,37, 44].
259 Overall, CagA was a comparable risk for both peptic ulcer disease and gastritis (OR 1.03,
260 95%CI: 0.37 to 2.91, 1763 participants, 13 studies, *I*^2^: 82%). There were no differences of the
261 risks in any subgroup of countries (Fig 3). Overall, presence of CagA was associated with an
262 increased risk of developing gastric cancer than gastritis (OR 2.53, 95%CI: 1.15 to 5.53, 613
263 participants, 7 studies, *I*^2^: 78%) (Fig 4), but a comparable risk for both gastric cancer and
264 peptic ulcer disease (OR 0.44, 95%CI: 0.08 to 2.34, 404 participants, 6 studies, *I*^2^: 67%) (Fig
265 5). Notably, in all these comparisons, there were substantial heterogeneities among the
266 studies.
267 267

### 268 Fig 3. Forest plot indicating the association of CagA with peptic ulcer

269 **disease**

### 270 Fig 4. Forest plot indicating the association of CagA in gastric cancer and

271 **peptic ulcer disease**

### 272 Fig 5. Forest plot indicating the association of CagA with gastric cancer

273 **and peptic ulcer disease**
274 274

## 275 Publication bias

276 A funnel showed fairly asymmetrical, indicating the presence of publication bias in
277 comparison between peptic ulcer disease and gastric cancer (13 studies included) (Fig 6).
278 Other comparisons (peptic ulcer diseases vs gastritis; gastric cancer vs gastritis) were not
279 possible to perform Eggers’s test as they had less than ten studies recommended for this
280 assessment.
281 281

### 282 Fig 6. Funnel plot asymmetry showing publication bias

283 283

# 284 Discussion

285 This study presents information on the pooled prevalence of CagA among those with *H.*
286 *pylori*-infected gastric disorders based on the data from 24 observational studies across eight
287 countries in the Indo-Pacific region. According to the findings, distinct *H. pylori* CagA
288 strains are circulating with geographical variation. The global prevalence of CagA-
289 positive *H. pylori* ranges from 43% to 90% [54]. and that was comparable with the current
290 findings of 82% in gastric disorders. After recalculation of data provided in a published
291 review including four Asian countries [55], CagA prevalence of 84% (95%CI: 67-100%) was
292 also comparable with the current findings, which encompassed 24 studies from eight Indo-
293 Pacific countries (82%, 95%CI: 72-99%).
294 Stomach mucosa in the antrum and corpus almost always becomes infiltrated by
295 neutrophilic and mononuclear cells during *H. pylori* colonization. The primary condition
296 associated with *H. pylori* colonization is chronic active gastritis, and additional conditions
297 specifically linked to *H. pylori* are the result of this continuing inflammatory process [56].
298 Hence, our analysis of the risk of developing peptic ulcer disease or gastric cancer compared
299 to gastritis is meaningful. Compared with gastritis, the presence of CagA had an increased
300 risk of developing gastric cancer observed in this study could be explained with biological
301 plausibility.
302 The difference in the occurrences of CagA of gastric cancer in India, Thailand, and
303 Malaysia may be explained by the phenomena of various strains circulating within different
304 ethnic groups, in conjunction with host and specific environmental factors. As described
305 elsewhere [57], *H. pylori* may have arrived in Asia as part of the five ancient human
306 migrations. The history of human population expansion in this region has been influenced by
307 multiple migrations and population expansions.
308 Molecular quantitative analysis showed that the load of CagA injected after one hour
309 was approximately 16% of the total CagA existing in the adjacent bacteria, and 30% after
310 three hours [58]. Taking into account of the low percentage of the total amount of bacteria in
311 the infection and the load of CagA injected by the bound bacteria, this might be one of many
312 reasons why there was low occurrence of gastric cancer in the countries with high incidence
313 of *H. pylori* infection.
314 CagA in the development of gastric disorder is not yet fully understood. There are
315 many ways in which translocated CagA interferes with host cell functions. For instance, the
316 CagA–PAR1 interaction [59], the interaction between SHP2 and gp130 (Src homology 2
317 domain-containing protein tyrosine phosphatase 2 and glycoprotein 130) [60], the interaction
318 of the IL6/gp130/STAT3 (interleukin-6/glycoprotein 130/signal transducer and activation of
319 transcription 3) pathway [60, 61], inflammation attribution to the changes in chemokine
320 profile of tissue [62], among others. Similar to previous meta-analyses [63,64], the current
321 study documented that CagA toxin of *H. pylori* may contribute to gastric cancer.

## 322 Study limitation

323 There are some limitations. This study contained solely published studies in English. As such,
324 there is an issue of information bias because it is likely to miss the unpublished studies or the
325 non-English publications that may have been appropriate. Regarding an inherent limitation of
326 cross-sectional/survey studies, the prevalence estimates in the primary studies identified for
327 the present analysis could vary as times go. Considering the associations observed in the
328 current meta-analysis, it would be crucial to substantiate the results using cohort studies with
329 extended follow-up times. The merit of observational studies is that they can determine health
330 problems in existent, reflecting real-life conditions. Due to the paucity of data, subgroups
331 such as the age of the study population, and unique types of gastric cancer (e.g., intestinal,
332 cardina, diffuse) could not be analysed. The present study included single genotype CagA.
333 Hence, selection bias is a concern. Future studies should consider multiple genotypes in
334 analysis. Due to limited number of studies that could compare CagA between the risks of
335 gastric cancer and peptic ulcer disease, a type II error may be an issue.
336 The estimated prevalence in this study with substantial heterogeneity might not be
337 related to sampling error only [65]. High heterogeneity may be observed albeit with more
338 homogenous subgroups such as countries. Therefore, a high *I^2^* value is not a signal that the
339 data are inconsistent [25]. Thus, these cautions should be considered while interpreting the
340 findings.

## 341 Implications

342 The higher prevalence of CagA in peptic ulcer disease reflects any intervention program for
343 *H. pylori* infection can benefit to reduction of the progression of gastritis to peptic ulcers.
344 Also, an increased risk of gastric cancer has implicated to requirement of effective
345 intervention that may reduce gastric cancer risk. As there were variations in frequency as well
346 as risk development among countries, reflecting variations in their ethnicities, a prioritization
347 of the high-risk population will be more cost-effective.
348 348
349 349
350 350

# 351 Conclusion

352 Findings suggest that among the eight countries in Indo-Pacific region, there is a varied
353 distribution of CagA in *H. pylori-*infected gastric disorders. There was a role of CagA in the
354 development of gastric cancer. To ensure the current findings of prevalence, and associations,
355 well-designed, large-sampled, prospective studies are recommended. If logistics allow,
356 studies from other locations and other virulent factors should be included in the future to
357 provide a more comprehensive global view.
358 358
359 359
360 360
361 361
362 362
363 363
364 364
365 365
366 366
367 367
368 368
369 369
370 370
371 371
372 372
373 373
374 374

# 375 Acknowledgements

376 We gratefully acknowledge the participants and authors of the primary studies that are
377 included in this study. We thank our institutions for allowing us to perform this study.
378 378
379 379
380 380
381 381
382 382
383 383
384 384
385 385
386 386
387 387
388 388
389 389
390 390
391 391
392 392

# 393 References

394 1. Chmiela M, Karwowska Z, Gonciarz W et al. Host pathogen interactions in *Helicobacter*
395 *pylori* related gastric cancer. World J Gastroenterol. 2017: 23, 1521-1540.
396 2. Marshall BJ, Warren JR (1984). Unidentified curved bacilli in the stomach of patients
397 with gastritis and peptic ulceration. Lancet.1984; 1:1311-1315.
398 3. IARC. Anonymous live flukes and *Helicobacter pylori.* IARC Working Group on the
399 Evaluation of Carcinogenic Risks to Humans, Lyon, 7–14 June 1994. IARC Monogr. Eval.
400 Carcinog. Risks Hum.1994; 61:1–241.
401 4. Lu H, Wu JY, Beswick EJ et al. Functional and intracellular signaling differences
402 associated with the *Helicobacter pylori* AlpAB adhesin from Western and East Asian strains.

403	J Biol Chem. 2007; 282: 6242-6254.

404

405	5. Crowe SE. *Helicobacter infection*, chronic inflammation, and the development of

406	malignancy. Curr Opin Gastroenterol*.*2005*:* 21:32–38.

407

408	6. Bakhti SZ, Latifi-Navid S, Safaralizadeh R. *Helicobacter pylori*-related risk predictors of

409	gastric cancer: The latest models, challenges, and future prospects. Cancer Med. 2020;

410	9(13):4808-4822.

411

412	7. Salama N, Guillemin K, McDaniel TK et al. A whole-genome microarray reveals genetic

413	diversity among *Helicobacter pylori* strains. Proc Natl Acad Sci*.* 2000; 97:14668–14673.

414

415	8. Selbach M, Moese S, Hauck CR, et al. Src is the kinase of the *Helicobacter pylori Cag*A

416	protein *in vitro* and *in vivo*. J Biol Chem. 2002; 277: 6775-6778.

417

418	9. Censini S, Lange C, Xiang Z, et al. *cag*, a pathogenicity island of *Helicobacter pylori*,

419	encodes type I-specific and disease-associated virulence factors. Proc. Natl. Acad. Sci.

420	U.S.A*.*1996*:* 93: 14648–14653.

421

422	10. Backert S, Tegtmeyer N, Selbach M. The versatility of *Helicobacter* pylori CagA effector

423	protein functions: The master key hypothesis. Helicobacter*.* 2010; 15:163–176.

424

425	11. Kwok T, Zabler D, Urman S et al. Helicobacter exploits integrin for type IV secretion and

426	kinase activation. Nature. 2007: 49:862-6.

427

428	12. Tegtmeyer N, Wessler S, Backert S. Role of the cag-pathogenicity island encoded type

429	IV secretion system in *Helicobacter pylori* pathogenesis. FEBS J. 2011: 278:1190-1202.

430

431	13. Yong X, Tang B, Li BS et al. *Helicobacter pylori* virulence factor *Cag*A promotes

432	tumorigenesis of gastric cancer via multiple signaling pathways. Cell Commun Signal. 2015:

433	13:30.

434

435	14. Higashi HA, Nakaya R, Tsutsumi K et al. *Helicobacter pylori Cag*A induces Ras-

436	independent morphogenetic response through SHP-2 recruitment and activation, J Biol

437	Chem. 2004: 79:17205-17216.

438

439	15. Miwa H, Go MF, Sato N.	*H. pylori* and gastric cancer: the Asian enigma. Am J

440	Gastroenterol.2002: 97(5):1106-1112.

441

442	16. Matsukura N, Yamada S, Kato S et al. Genetic differences in interleukin-1 beta

443	polymorphisms among four Asian populations: an analysis of the Asian paradox between *H.*

444	*pylori* infection and gastric cancer incidence. J Exp Clin Cancer Res. 2003: 22:47-55.

445

446	17. GLOBOCAN: [50-bangladesh-fact-sheets.pdf (iarc.fr)](https://gco.iarc.fr/today/data/factsheets/populations/50-bangladesh-fact-sheets.pdf)

447	gco.iarc.fr/today/data/factsheets/populations/50-bangladesh-fact-sheets.pdf 2020

448

449	18. Hooi JKY, Lai WY, Ng WK et al. Global prevalence of *Helicobacter pylori* infection:

450	systematic review and meta-analysis. Gastroenterology. 2017: 153:420-429.

451

452	19. Kakinuma D, Arai H, Yasuda T et al. Treatment of gastric cancer in Japan. J Nippon Med

453	Sch. 2021; 8:156-162.

454

455	20. Yang L, Kartsona C, Yao P et al. The relative and attributable risks of cardia and non-

456	cardia gastric cancer associated with *Helicobacter pylori* infection in China: a case-cohort

457	study. Lancet Public Health. 2021: e888-896.

458

459	21. Park SH, Kang MJ, Yun EH, Jung KW. Epidemiology of gastric cancer in Korea: Trends

460	in incidence and survival based on Korea Central Cancer Registry Data (1999-2019). J

461	Gastric Cancer. 2022; 22:160-168.

462

463	22. Page MJ, McKenzie J E, Bossuyt PM et al. The PRISMA 2020 statement: an updated

464	guideline for reporting systematic reviews. BMJ. 2021; 372, n71.

465

466	23. Zheng TH, Zhao JL, Guleng B. Advances in Molecular Biomarkers for Gastric Cancer.

467	Crit Rev Eukaryot Gene Expr.2015: 25:299-305.

468

469	24. Wells G, Shea B, O’Connell D et al (2014). The Newcastle-Ottawa Scale (NOS) for

470	assessing the quality of nonrandomised studies in meta-analyses.

471	https:/[/www](http://www.semanticscholar.org/paper/The-Newcastle-Ottawa-Scale-(NOS)).[semanticscholar.org/paper/The-Newcastle-Ottawa-Scale-(NOS)](http://www.semanticscholar.org/paper/The-Newcastle-Ottawa-Scale-(NOS))

472

473	25. Barker TH, Migliavaca CB, Stein C et al. Conducting proportional meta-analysis in

474	different types of systematic reviews: a guide for synthesisers of evidence. BMC Med Res

475	Methodol.2021; 21, 189.

476

477	26. Nyaga VN, Arbyn M, Aerts M. [METAPROP_ONE: Stata module to perform fixed and](https://ideas.repec.org/c/boc/bocode/s457861.html)

478	[random effects meta-analysis of proportions](https://ideas.repec.org/c/boc/bocode/s457861.html). [Statistical Software Components](https://ideas.repec.org/s/boc/bocode.html) S457861,

479	Boston College Department of Economics, revised 26 Jan 2017.

480

481	27. DerSimonian R, Laird N. Meta-analysis in clinical trials. Control Clin. Trials. 1986; 7,

482	177-188.

483

484 28. Deeks JJ, Higgins JP, Altman DG, editor(s). Chapter 10: Analysing data and undertaking
485 meta-analyses. In: Higgins JP, Thomas J, Chandler J, Cumpston M, Li T, Page MJ, Welch
486 VA (editors). Cochrane Handbook for Systematic Reviews of Interventions Version 6.3
487 (updated February 2022). Cochrane, 2022. Available from training.cochrane.org/handbook.
488 29. Egger M, Davey Smith G, Schneider M, Minder C. Bias in meta-analysis detected by a
489 simple, graphical test. BMJ. 1997: 315:629-364.
490 490
491 30. Alfizah,H, Ramelah M, Rizal AM, et al. Association of Malaysian *Helicobacter pylori*
492 virulence polymorphisms with severity of gastritis and patients' ethnicity. Helicobacter. 2012;

493	17:340-349.

494

495	31. Ali M, Khan AA, Tiwari SK, et al. Association between cag-pathogenicity island in

496	*Helicobacter pylori* isolates from peptic ulcer, gastric carcinoma, and non-ulcer dyspepsia

497	subjects with histological changes. World J Gastroenterol 2005; 11(43):6815-6822.

498

499	32. Ansari S, Kabamba ET, Shrestha PK, et al. *Helicobacter pylori* bab characterization in

500	clinical isolates from Bhutan, Myanmar, Nepal and Bangladesh. PLoS One. 2017;

501	12:e0187225.

502

503	33. Boonyanugomol W, Kongkasame W, Palittapongarnpim P et al. Genetic variation in the

504	cag pathogenicity island of *Helicobacter pylori* strains detected from gastroduodenal patients

505	in Thailand. Braz J Microbiol. 2020; 51:1093-1101.

506

507	34. Chomvarin C, Namwat W, Chaicumpar K et al. Prevalence of *Helicobacter pylori* vacA,

508	cagA, cagE, iceA and babA2 genotypes in Thai dyspeptic patients. Int J Infect Dis.2008; 12:

509	30-36.

510

511	35. Chomvarin C, Phusrim K, Sawadpanich K et al. Prevalence of cagA EPIYA motifs in

512	*Helicobacter pylori* among dyspeptic patients in northeast. Southeast Asian J Trop Med

513	Public Health. 2012; 43:105-115.

514

515	36. Jeyamani L, Jayarajan J, Leelakrishnan V, Swaminathan M. *CagA* and *VacA* genes

516	of *Helicobacter pylori* and their clinical relevance. Indian J Pathol Microbiol. 2018; 61(1):

517	66-69.

518

519	37. Linpisarn S, Suwan W, Lertprasertsuk N. *Helicobacter pylori* cagA, vacA and iceA

520	genotypes in northern Thai patients with gastric disease. Southeast Asian J Trop Med Public

521	Health. 2007; 38(2):356-362.

522

523	38. Miftahussurur M, Syam AF, Makmun D et al. *Helicobacter pylori* virulence genes in the

524	five largest islands of Indonesia. Gut Pathog*.* 2015; 7:26.

525

526	39. Mohamed R, Hanafiah A, Rose IM et al. *Helicobacter pylori* cagA gene variants in

527	Malaysians of different ethnicity. Eur J Clin Microbiol Infect Dis. 2009; 28:865–869.

528

529	40. Myint T, Miftahussurur M, Vilaichone RK et al. Characterizing *Helicobacter pylori*

530	*cagA* in Myanmar. Gut Liver. 2018; 12:51-57.

531

532 41. Nguyen TL, Uchida T, Tsukamoto Y et al. *Helicobacter pylori* infection and
533 gastroduodenal	diseases	in	Vietnam: a	cross-sectional, hospital-based	study.	BMC
534 Gastroenterol.2010; 10:114
535 42. Nguyen TC, Tang NLC, Le GKN, et al. *Helicobacter pylori* infection and peptic ulcer
536 disease in symptomatic children in southern Vietnam: a prospective multicenter study.

537	Healthcare.2023; 11, 1658.

538

539	43. Pandya HB, Agravat HH, Patel JS. Prevalence of Specific *Helicobacter Pylori cagA,*

540	*vacA, iceA, ureC* genotypes and its clinical relevance in the patients with acid-peptic diseases.

541	J Clin Diagn Res.2017; 11(8): DC23-DC26.

542

543	44. Schmidt HM, Goh KL, Fock KM et al. Distinct cagA EPIYA motifs are associated with

544	ethnic diversity in Malaysia and Singapore. Helicobacter. 2009; 14:256–263.

545

546	45. Schmidt HM, Andres S, Nilsson C et al. The cagA PAI is intact and functional but

547	hp0520 varies significantly in *Helicobacter pylori* isolates from Malaysia and Singapore. Eur

548	J Clin Microbiol Infect Dis.2010; 29:439–451.

549

550	46. Tan HJ, Rizal AM, Rosmadi MY, Goh KL. Distribution of *Helicobacter pylori* cagA,

551	cagE and vacA in different ethnic groups in Kuala Lumpur, Malaysia. J Gastroenterol

552	Hepatol. 2005; 20:589–594.

553

554	47. Tan HJ, Rizal AM, Rosmadi MY, Goh KL. Role of *Helicobacter pylori* virulence factor

555	and genotypes in non-ulcer dyspepsia. J Gastroenterol Hepatol.2006; 21: 110-111.

556

557	48. Tiwari SK, Sharma V, Sharma VK et al. Phylogenetic analysis, based on EPIYA repeats

558	in the CagA gene of Indian *Helicobacter pylori*, and the implications of sequence variation in

559	tyrosine phosphorylation motifs on determining the clinical outcome. Genet Mol Biol. 2011;

560	34: 280-285.

561

562	49. Trang TT, Shiota S, Matsuda M et al. The prevalence of *Helicobacter pylori* virulence

563	factors in Bhutan, Vietnam, and Myanmar is related to gastric cancer incidence. Biomed Res

564	Int. 2015; 830813.

565

566	50. Truong BX, Mai VT, Tanaka H et al. Diverse characteristics of the cagA gene of

567	*Helicobacter pylori* strains collected from patients from South Vietnam with gastric cancer

568	and peptic ulcer. J Clin Microbiol. 2009; 47: 4021–4028.

569

570	51. Uchida T, Nguyen LT, Takayama A et al. Analysis of virulence factors of *Helicobacter*

571	*pylori* isolated from a Vietnamese population. BMC Microbiol. 2009; 9:175.

572

573	52. Yamada S, Kato S, Matsuhisa T et al. Predominant mucosal IL-8 mRNA expression in

574	non-cagA Thais is risk for gastric cancer. World J Gastroenterol. 2013; 19(19):2941-9.

575

576	53. Zheng PY, Hua J, Yeoh KG, Ho B. Association of peptic ulcer with increased expression

577	of Lewis antigens but not cagA, iceA, and vacA in *Helicobacter pylori* isolates in an Asian

578	population. Gut.2000; 47:18–22.

579

580	54. Li Y, Choi H, Leung K, et al. Global prevalence of *Helicobacter pylori* infection between

581	1980 and 2022: a systematic review and meta-analysis. Lancet Gastroenterol Hepatol. 2023;

582	8(6):553-564.

583	55. Sahara S, Sugimoto M, Vilaichone RK et al. Role of *Helicobacter pylori* cagA EPIYA

584	motif and vacA genotypes for the development of gastrointestinal diseases in Southeast Asian

585	countries: a meta-analysis. BMC Infect Dis. 2012; 12:223.

586

587	56. Kusters JG, van Vliet AH, Kuipers EJ. Pathogenesis of *Helicobacter pylori* infection.

588	Clin Microbiol Rev.2006; 19:449-490.

589

590	57.	Breurec	S,	Guillard	B,	Hem	S	et	al.	Evolutionary	History	of *Helicobacter*

591	*pylori* Sequences reflect past human migrations in Southeast Asia. Plos One.2011; 6(7):

592	e22058.

593

594	58. Jimenez-Soto LF, Haas R. The CagA toxin of *Helicobacter pylori*: abundant production

595	but relatively low amount translocated. Sci Rep. 2016; 6:23227

596

597	59. Saadat I, Higashi H, Obuse C. et al*. Helicobacter pylori* CagA targets PAR1/MARK

598	kinase to disrupt epithelial cell polarity. Nature.2007; 447, 330-333.

599

600	60. Lee IO, Kim JH, Choi YJ, et al. *Helicobacter pylori* CagA phosphorylation status

601	determines the gp130-activated SHP2/ERK and JAK/STAT signal transduction pathways in

602	gastric epithelial cells. J Biol Chem. 2010; 285:16042-16050.

603

604	61. Ohtani T, Ishihara K, Atsumi T, et al. Dissection of signaling cascades through gp130 in

605	vivo: reciprocal roles for STAT3- and SHP2-mediated signals in immune responses.

606	Immunity. 2000; 12:95-105

607

608	62. Posselt G, Backert S, Wessler S. The functional interplay of *Helicobacter pylori* factors

609	with gastric epithelial cells induces a multi-step process in pathogenesis. Cell Commun

610	Signal. 2013; 11:77.

611

612	63. Matos JI, de Sousa HA, Marcos-Pinto R, Dinis-Ribeiro M. *Helicobacter pylori Cag*A and

613	*Va*cA genotypes and gastric phenotype: a meta-analysis. Eur J Gastroenterol Hepatol. 2013;

614	25:1431-1441.

615

616	64. Pormohammad A, Mohtavinejad N, Gholizadeh P, et al. Global estimate of gastric cancer

617	in Helicobacter pylori-infected population: A systematic review and meta-analysis. J Cell

618	Physiol. 2019; 234:1208-1218.

619

620	65. Barendregt JJ, Doi SA, Lee YY, et al. Meta-analysis of prevalence. J. Epidemiol.

621	Community Health.2013; 67, 974–978.

622

623

624

625

626

627

628

629

630

631

632

# 633 Supplementary Information

634 S1 Checklist. PRISMA-2020 checklist
635 S 1 Table. Search Strategy
636 S 2 Table. Excluded studies and the reasons for exclusion
637 S 3 Table. Quality assessment with the NOS assessment criteria.
638 S1 Fig. Pooled prevalence of CagA in *H. pylori-* infected gastritis.
639 S2 Fig. Pooled prevalence of CagA in *H. pylori-* infected peptic ulcer disease.
640 S3 Fig. Pooled prevalence of CagA in *H. pylori-* infected gastric cancer
641 641

Figure 4	[Click here to access/download;Figure;fig-4	.tiff](https://www2.cloud.editorialmanager.com/pone/download.aspx?id=36115431&guid=b323a3a4-5bd4-47eb-9a77-e5c61608830e&scheme=1)

S1 Checklist

#### [Click here to access/download](https://www2.cloud.editorialmanager.com/pone/download.aspx?id=36027379&guid=89860577-17b9-4cd1-af7d-68c34fbf5913&scheme=1)

[**Supporting Information** S1 Checklist_.doc](https://www2.cloud.editorialmanager.com/pone/download.aspx?id=36027379&guid=89860577-17b9-4cd1-af7d-68c34fbf5913&scheme=1)

#### [Click here to access/download](https://www2.cloud.editorialmanager.com/pone/download.aspx?id=36027381&guid=94dea6fb-8f82-4840-b9c6-52c18bdfda86&scheme=1)

[**Supporting Information** S1 Table_.doc](https://www2.cloud.editorialmanager.com/pone/download.aspx?id=36027381&guid=94dea6fb-8f82-4840-b9c6-52c18bdfda86&scheme=1)

#### [Click here to access/download](https://www2.cloud.editorialmanager.com/pone/download.aspx?id=36115325&guid=f78df9d7-db92-497d-ae92-cb77d668bc7a&scheme=1)

[**Supporting Information** S2 Table	.doc](https://www2.cloud.editorialmanager.com/pone/download.aspx?id=36115325&guid=f78df9d7-db92-497d-ae92-cb77d668bc7a&scheme=1)

#### [Click here to access/download](https://www2.cloud.editorialmanager.com/pone/download.aspx?id=36115326&guid=8da9e383-25f6-4399-8490-1ac237c13b1a&scheme=1)

[**Supporting Information** S3 Table_.doc](https://www2.cloud.editorialmanager.com/pone/download.aspx?id=36115326&guid=8da9e383-25f6-4399-8490-1ac237c13b1a&scheme=1)

#### [Click here to access/download](https://www2.cloud.editorialmanager.com/pone/download.aspx?id=36027386&guid=18a60809-11db-4cb8-80ba-bed1c34c2cfb&scheme=1)

[**Supporting Information** S1 Fig.doc](https://www2.cloud.editorialmanager.com/pone/download.aspx?id=36027386&guid=18a60809-11db-4cb8-80ba-bed1c34c2cfb&scheme=1)

#### [Click here to access/download](https://www2.cloud.editorialmanager.com/pone/download.aspx?id=36027390&guid=b7e44683-e0fb-4a0f-86b1-23fe70763956&scheme=1)

[**Supporting Information** S2 Fig.doc](https://www2.cloud.editorialmanager.com/pone/download.aspx?id=36027390&guid=b7e44683-e0fb-4a0f-86b1-23fe70763956&scheme=1)

#### [Click here to access/download](https://www2.cloud.editorialmanager.com/pone/download.aspx?id=36027391&guid=2c0f7819-250d-4899-9cdc-9409d3682b62&scheme=1)

[**Supporting Information** S3 Fig.doc](https://www2.cloud.editorialmanager.com/pone/download.aspx?id=36027391&guid=2c0f7819-250d-4899-9cdc-9409d3682b62&scheme=1)
