# Supplementary material for: CagA toxin and risk of Helicobacter pylori-infected gastric phenotype: A meta-analysis of observational studies
Source: PLoS One. 2024 Aug 22;19(8):e0307172. doi: 10.1371/journal.pone.0307172 (PMC11341061; doi:10.1371/journal.pone.0307172)
Supplement: Attachment — Submitted filename: Authors responses to the comments_ 170624.doc [file pone.0307172.s009.doc]

**CagA toxin and risk of Helicobacter pylori-infected gastric phenotype: A meta-analysis of observational studies**

Reviewer #1: Dear Editorial office

It is my honour to read and review this nicely entitled paper by salomon et al “CagA toxin and risk of Helicobacter pylori-infected gastric phenotype: A meta-analysis of observational studies” submitted to Plos one. In principle I found it suitable for publication here pending your corrections.

Q1- The title seems fine and no change warranted.

**Authors response**

Thank you.

Q2- Abstract is too general-like, so please enrich it

**Authors response**

We have improved this section**.**

**In the revised version**

**Background**

*Helicobacter pylori* (*H.pylori*) is frequently associated with non-cardia type gastric cancer, and it is designated as a group I carcinogen. This study aimed to systematically review and meta-analyse the evidence on the prevalence of CagA status in people with gastric disorders in the Indo-Pacific region, and to examine the association of CagA positive in the risk of gastric disorders. This study focused on the Indo-Pacific region owing to the high disability adjusted life-years related to these disorders, the accessibility of efficient treatments for this common bacterial infection, and the varying standard of care for these disorders, particularly among the elderly population in the region.

**Methods**

Relevant studies were identified in the health-related electronic databases including PubMed, Ovid, Medline, Ovid Embase, Index Medicus, and Google Scholar that were published in English between January 1, 2000, and November 18, 2023. For pooled prevalence, meta-analysis of proportional studies was done, after Freeman-Tukey double arcsine transformation of data. A random-effect model was used to compute the pooled odds ratio (OR) and 95% confidence interval (CI) to investigate the relationship between CagA positivity and gastric disorders.

**Results**

Twenty-four studies from eight Indo-Pacific countries (Bhutan, India, Indonesia, Malaysia, Myanmar, Singapore, Thailand, Vietnam) were included. Overall pooled prevalence of CagA positivity in *H. pylori-*infected gastric disorders was 83% (73-91%). Following stratification, the pooled prevalence of CagA positivity was 78% (95%CI:67-90%) in *H. pylori*-infected gastritis, 86% (95%CI = 73-96%) in peptic ulcer disease, and 83% (95%CI = 51-100%) in gastric cancer. Geographic locations encountered variations in CagA prevalence. There was a greater risk of developing gastric cancer in those with CagA positivity compared with gastritis (OR = 2.53,95%CI = 1.15-5.55).

**Conclusion**

Findings suggest that the distribution of CagA in *H. pylori-*infected gastric disorders varies among different type of gastric disorders in the study countries, and CagA may play a role in the development of gastric cancer. It is important to provide the high standard of care for the management of gastric diseases, particularly in a region where the prevalence of these disorders is high. Better strategies for effective treatment for high-risk groups are required for health programs to revisit this often-neglected infectious disease.

Q3- Abstract “To confirm these findings, well-designed studies with sufficient samples are recommended.” Is not absolutely a good text for here, remove it.
**Authors response**

We have removed it from the revised version. Thank you.

Q4- Page 4, line 76, why there are two major types of reference methods? “epithelial cells [4],4 which” revise it for revision.

**Authors response**

It was a typing error. We have corrected it. Thank you**.**

Q5- Introduction section sentence “This reflects that, in
addition to its urease activity, H. pylori possesses additional virulence factors that are required to enable it to colonize and survive in the stomach's acid environment.” We need the below text references

** Abadi, A. T. B. (2017). Strategies used by helicobacter pylori to establish persistent infection. World journal of Gastroenterology, 23(16), 2870.
*** Israel, Dawn A., and Richard M. Peek Jr. "The role of persistence in Helicobacter pylori pathogenesis." Current opinion in gastroenterology 22.1 (2006): 3-7.
**Authors response**

Thank you. We have added these two references (Ref# 4, 5)**.**

**In the revised version**

Q6- Prisma check list and other related files are fine and I have no word. The total writing style in this paper is good actually.

**Authors response**

Thank you.

Q7- Publication bias and its likely impact should be well-indicated in the revised version.
**Authors response**

We have provided an issue regarding publication bias along with a reference.

**In the revised version**

**Study limitation**

Due to publication bias related to small studies that tended to exaggerate the true effect [65], as well as limited number of studies that could compare CagA between the risks of gastric cancer and peptic ulcer disease, a type II error may be an issue.

**Reference**

65. Newcombe RG. Towards a reduction in publication bias. Br Med J (Clin Res Ed). 1987;295(6599):656-659

Q8- While i am reading the discussion, I cannot find the exact novelty of this research although I know how precious IS THIS research. Please indicate all novelty points of this research in new version.

**Authors response**

Agreed. There is not novelty in methodology. However, a specific evidence based on data from specific endemic countries in a particular region is informative. We have intended to provide a specific information of the targeted region. Hence, we have added these concerns in “implications”.

**In the revised version**

**Implications**

Reiterating the substantial evidence of association between a treatable infection and a high burden of disease and health care utilisation can help improve equity in their programs during this second half of the Sustainable Development Goal period, when countries in the region are struggling to attain the universal health coverage goal set [20].

Q9- Some of the references are outdated, please revise them all.
**Authors response**

Thank you. We have used cotemporary references as well as a few old references to honour the original authors. References published in 2023 are also included.

Q10- Hpylori should be italic throughout the paper.
**Authors response**

We have corrected it. Thank you.

Q11- A minor polish for English is also needed.

**Authors response**

We have improved language proficiency in this revised version. Thank you.

Reviewer #2: Dear authors,

This is a meta-analysis dealing with the prevalence of CagA toxin and risk of Helicobacter pylori-infected gastric phenotype in Indo-Pacific region. The authors came to the conclusion that the distribution of CagA in Helicobacter pylori-infected gastric disorders varies throughout the eight Indo-Pacific countries, and CagA has a role in the development of gastric cancer.

The article is well-written however it does not add any new findings to the well known evidence regarding the relationship of *Helicobacter pylori* infection and gastric cancer.

**Authors response**

Thank you. We have intended to provide a specific information of the targeted region. We have noted in the revision that this study focused on people in the Indo-Pacific countries given the high DALY burden attributed to these disorders, the costs of management to the health system, the availability of effective treatments of this common bacterial infection and the variable quality of care for these disorders especially amongst older people in the region. We have added these points under “implication”.

Reviewer #3: I congratulate the authors for a good work, though it requires some modifications which I have included in the manuscript. Authors should also revise the introduction and make it a background and not sort of discussion.

**Authors response**

We have done corrections, following the points shown in the Manuscript.

We have improved the introduction, as suggested. Thank you.

**Comments shown in the manuscript file of PONE-D-24-13758**

Thank you for the comments and inputs provided. Below are the authors responses and subsequent texts in the revised version.

Q/1

The aims of the study don’t line up with the title of the study

**Authors response**

We have rephrased the objectives to be clearer. We feel that the title is fit to the revised objectives.

**In the revised version**

**Abstract**

**Background**

This study aimed to systematically review and meta-analyse the evidence on the prevalence of CagA status in people with gastric disorders in the Indo-Pacific region, and to examine the association of CagA positive in the risk of gastric disorders.

Q2/ Specify them

It should also be specified the time interval for the studies included in the study

**Authors response**

We have provided, accordingly.

**In the revised version**

**Search strategy**

Relevant studies were identified in the health-related electronic databases including PubMed, Ovid, Medline, Ovid Embase, Index Medicus, and Google Scholar that were published in English between 1 January 2000, and 18 November 2023.

Q3/

I suggest authors should use the word “pooled” instead of “summary

**Authors response**

We have updated, as suggested.

**In the revised version**

For pooled prevalence, meta-analysis of proportional studies was done, after Freeman-Tukey double arcsine transformation of data.

Q 4/Not disorders?

**Authors response**

We have corrected it, accordingly.

**In the revised version**

A random-effect model was used to compute the summary odds ratio (OR) and 95%

confidence interval (CI) to investigate the relationship between CagA and gastric

disorders.

Q5/

This should be termed as “overall pooled prevalence

**Authors response**

We have updated, accordingly.

**In the revised version**

Overall pooled prevalence of CagA positivity in *H. pylori-*infected gastric disorders was 83% (95% CI=73-91%).

Q6/ This sentence must be rephrased. Use = symbol and not : symbol

Compared with gastritis, there was a greater risk of developing gastric cancer in

those with CagA positivity (OR:2.53,95%CI:1.15-5.55)

**Authors response**

We have updated this part.

**In the revised version**

There was a greater risk of developing gastric cancer in those with CagA positivity compared with gastritis (OR = 2.53,95%CI =1.15-5.55).

Q7/ Conclusion

But this is not what is shown in the result section of the abstract. The variation of CagA positivity is not for countries but for different gastric disorders.

This information can’t be taken as a direct association. Authors should show plausibility and not causal-effect relationship

**Authors response**

Accordingly, we have updated this part. We have deleted the last sentence, following a comment by the Reviewer # 1.

**In the revised version**

Findings suggest that the distribution of CagA in *H. pylori-*infected gastric disorders varies among different types of gastric disorders in the study countries, and CagA may play a role in the development of gastric cancer.

Q 8/ These keywords cant help in searching of the work

**Authors response**

We have updated, accordingly.

**In the revised version**

**Keywords:**

*Helicobacter pylori*, CagA, gastritis, gastric cancer, peptic ulcers, meta-analysis, Indo-Pacific

Q9/

Introduction....... This abbreviation must first be named before being used.

**Authors response**

We have provided, accordingly.

**In the revised version**

*Helicobacter pylori* (*H.pylori*) is a gram-negative bacterium that colonizes the stomach and is attributed to causing infections in humans [1.2].

Q 10/ Introduction

This information then contradicts with the statement given in the conclusion part of the abstract. After all, the number of gastritis or PUD is higher than that of gastric cancer. Although both have high prevalence *of H. pylori*.

**Authors response**

We have updated the abstract. Taking together with the reviewer #1’s comment, we have deleted the contradictory sentence in the abstract.

Q11/

Taken together………….what do you mean?

**Authors response**

We wish to say that taking all factors into consideration. We have rephrased this phrase in the revised version.

**Authors response**

We have rephrased the objectives.

**In the revised version**

Taking all of these into consideration, the objectives of this study were to systematically review and meta-analyse the evidence on the prevalence of CagA status in people with gastric disorders in the Indo-Pacific region, and to examine the association of CagA positive in the risk of gastric disorders.

Q12/ Materials and Methods

what? This study sticked to the PRISMA-2020 checklist [22] [S1 Checklist].

The protocol of this……………Move this information to the declarations.

**Authors response**

We have replaced with a term “followed”. Thank you.

**In the revised version**

**Materials and Methods**

- This study followed the PRISMA-2020 checklist [22] [S1 Checklist].

**Q13/ Search strategy**

Correct the use of punctuation marks

**Authors response**

We have done this, accordingly. Thank you.

**Q14/Selection criteria**

Did authors take into consideration issue of homogeneity for the included studies based on similarity of the detection methods of CagA?

This is because all detection methods don’t have the same detection capacity

**Authors response**

We have considered studies regardless of the CagA detection methods.

**In the revised version**

**Materials and Methods**

**Selection criteria**

3. Assessed the CagA status, irrespective of the detection method.

Q15/

What is the message? Is there another way of considering this?

**Authors response**

We wish to say that we have followed the type of disorders according to the primary study. **In the revised version**

**Materials and Methods**

**Selection criteria**

In this study, the participants with duodenal and/or gastric ulcerations were categorised as peptic ulcer disease as stated in the primary studies.

Q16/ Data collection

Check grammar. How? This is not clear

**Authors response**

We have rephrased this part.

**In the revised version**

Full-text studies that deemed appropriate were further reviewed. Any discrepancy between the two investigators was settled by agreement.

Q17/ Table 2 Place references in column 2

**Authors response**

We have updated the table columns.

**In the revised version**

Table 1, 2

Q17/ Include reference

**Authors response**

We have provided a reference.

**In the revised version**

Stomach mucosa in the antrum and corpus almost always becomes infiltrated by neutrophilic and mononuclear cells during *H. pylori* colonization [56].

**References**

56. Kusters JG, van Vliet AH, Kuipers EJ. Pathogenesis of *Helicobacter pylori* infection. Clin Microbiol Rev.2006; 19:449-490.

xxxxxxxxxxxxxxxxxxxxxxxxxxxxxxxxxxxxxxxxxxxxxxxx

Journal requirements:

When submitting your revision, we need you to address these additional requirements.

1. Please ensure that your manuscript meets PLOS ONE's style requirements, including those for file naming. The PLOS ONE style templates can be found at

<https://journals.plos.org/plosone/s/file?id=wjVg/PLOSOne_formatting_sample_main_body.pdf> and

<https://journals.plos.org/plosone/s/file?id=ba62/PLOSOne_formatting_sample_title_authors_affiliations.pdf>

2. We note that your Data Availability Statement is currently as follows: [All relevant data are within the manuscript and its Supporting Information files.]

Please confirm at this time whether or not your submission contains all raw data required to replicate the results of your study. Authors must share the “minimal data set” for their submission. PLOS defines the minimal data set to consist of the data required to replicate all study findings reported in the article, as well as related metadata and methods ([https://journals.plos.org/plosone/s/data-availability#loc-minimal-data-set-definition](https://journals.plos.org/plosone/s/data-availability#loc-minimal-data-set-definition)).

For example, authors should submit the following data:

- The values behind the means, standard deviations and other measures reported;

- The values used to build graphs;

- The points extracted from images for analysis.

Authors do not need to submit their entire data set if only a portion of the data was used in the reported study.
